# Major Facilitator Superfamily Domain-Containing Protein 2a (MFSD2A) Has Roles in Body Growth, Motor Function, and Lipid Metabolism

**DOI:** 10.1371/journal.pone.0050629

**Published:** 2012-11-29

**Authors:** Justin H. Berger, Maureen J. Charron, David L. Silver

**Affiliations:** 1 Department of Biochemistry, Albert Einstein College of Medicine, Bronx, New York, United States of America; 2 Department of Medicine, Division of Endocrinology, Albert Einstein College of Medicine, Bronx, New York, United States of America; 3 Department of Obstetrics and Gynecology and Women’s Health, Albert Einstein College of Medicine, Bronx, New York, United States of America; 4 Signature Research Program in Cardiovascular & Metabolic Disorders, Duke-NUS Graduate Medical School, Singapore, Singapore; Nihon University School of Medicine, Japan

## Abstract

The metabolic adaptations to fasting in the liver are largely controlled by the nuclear hormone receptor peroxisome proliferator-activated receptor alpha (PPARα), where PPARα upregulates genes encoding the biochemical pathway for β-oxidation of fatty acids and ketogenesis. As part of an effort to identify and characterize nutritionally regulated genes that play physiological roles in the adaptation to fasting, we identified *Major facilitator superfamily domain-containing protein 2a* (Mfsd2a) as a fasting-induced gene regulated by both PPARα and glucagon signaling in the liver. MFSD2A is a cell-surface protein homologous to bacterial sodium-melibiose transporters. Hepatic expression and turnover of MFSD2A is acutely regulated by fasting/refeeding, but expression in the brain is constitutive. Relative to wildtype mice, gene-targeted Mfsd2a knockout mice are smaller, leaner, and have decreased serum, liver and brown adipose triglycerides. Mfsd2a knockout mice have normal liver lipid metabolism but increased whole body energy expenditure, likely due to increased β-oxidation in brown adipose tissue and significantly increased voluntary movement, but surprisingly exhibited a form of ataxia. Together, these results indicate that MFSD2A is a nutritionally regulated gene that plays myriad roles in body growth and development, motor function, and lipid metabolism. Moreover, these data suggest that the ligand(s) that are transported by MFSD2A play important roles in these physiological processes and await future identification.

## Introduction

The rampant obesity epidemic has led to interrelated increases in cardiovascular disease, hepatic steatosis, and diabetes [1]–[5]. A better understanding of how the body uses excess nutrients and switches between anabolic and catabolic states is crucial to advance clinical treatments. The nuclear hormone receptor PPARα acts as a master regulator, along with adrenergic and hormonal signals, to increase fatty acid oxidation (FAO) [6] and gluconeogenesis [7], as well as ketogenesis during the latter stages of a fast (>12 h) [8],[9]. The PPARα knockout (KO) mouse constructed by Gonzalez and colleagues proved the importance of PPARα in coordinating the transcriptional response to fasting in liver [10]. FAO in brown adipose tissue (BAT) is also regulated in a PPARα-dependent manner and promotes non-shivering thermogenesis via uncoupled oxidative phosphorylation in response to adrenergic and hormonal (particularly thyroid) signals [11], [12]. With the recent demonstration of BAT in adult humans, this pathway has gained significance in its potential for therapeutic intervention [13], [14].

In an effort to understand the physiological processes that govern the metabolic transition from fed to fasted state, we used a microarray approach to identify novel PPARα targets in liver [15]. From this screen we identified the plasma membrane protein, *Major facilitator superfamily domain-containing protein 2a* (Mfsd2a). The Major Facilitator Superfamily (MFS) is one of the largest families of transporters, consisting of 74 functionally diverse subfamilies, with ligands ranging from simple and complex sugars and amino acids to drugs and organic anions [16]. The majority of MFS porters share a 12-transmembrane domain homology made of two evolutionary duplicated 6-transmembrane units. Mfsd2a is phylogenetically conserved from fish to human, and a recent study reported a predicted structure of 12 transmembrane domains with a 29% identity to the bacterial sodium-melibiose transporter, MelB [17].

Human, but not mouse, MFSD2A was also identified as the target for syncytin-2, where syncytin-2 binding to MFSD2A induces cell fusion and thus likely plays an important role in syncytiotrophoblast development in human placenta [18]. Additionally Angers et al. reported *Mfsd2a* mRNA is induced in mouse liver by fasting and in BAT by fasting, cold exposure, and β-adrenergic signaling, indicating a potential physiological role in lipid metabolism and energy expenditure [17].

In the current study, our goal was to determine the regulatory pathways that control Mfsd2a expression in response to fasting, and to determine a physiological role for MFSD2A using a gene-targeted Mfsd2a-deficient mouse model. We provide evidence that MFSD2A plays an important role in regulating lipid metabolism, growth and development, and motor function, and suggests that the ligand(s) transported by MFSD2A are important in metabolism.

**Figure 1:**
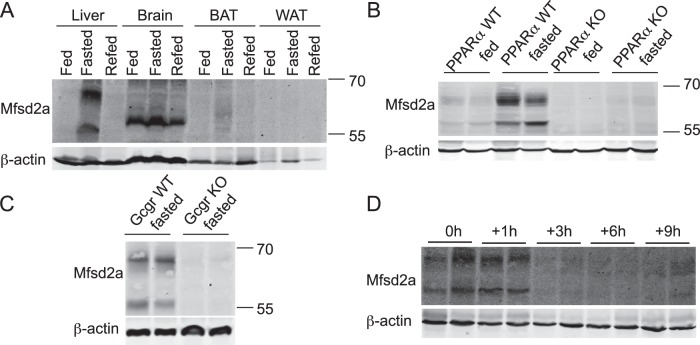
MFSD2A expression is nutritionally regulated and requires PPARα and glucagon signaling. *A,* Representative Western blot of MFSD2A expression in the indicated tissues isolated from WT mice that were *ad libitum* fed, fasted for 24 h, or fasted for 24 h and *ad libitum* refed (n = 3 mice per condition). *B*, Western blot of liver samples from fed and fasted wildtype (WT) and PPARα knockout (KO) mice (n = 4 per genotype per condition). *C*, Western blot of livers from two glucagon receptor (Gcgr KO)-deficient mice and two WT fasted littermates probed for MFSD2A expression (n = 6–7). *D*, Western blot for MFSD2A from liver samples of 24 h fasted WT mice (0 h), and mice fasted, refed and sacrificed 1 to 9 h after refeeding. β-actin served as a loading control in all panels. Each lane represents a liver sample from an individual animal.

## Materials and Methods

### Antibodies and Plasmids

A polyclonal MFSD2A antibody was raised in rabbits against the c-terminal peptide H-CSDTDSTELASIL-OH (NeoMPS, San Diego, CA). Serum was purified on polyA/G resin followed by affinity purification against the peptide immobilized on Sulfolink resin (ThermoScientific). Other antibodies used in these studies include: β-actin (Sigma), Tom20 (Santa Cruz), Cox4 (Cell Signal), and UCP1 (Abcam). Mouse and human Mfsd2a cDNA was subcloned into pcDNA3.1 (Invitrogen).

### Cell Culture

HEK293 cells (ATCC) were cultured in DMEM (Invitrogen) with 10% fetal bovine serum and penicillin/streptomycin at 37°C with 5% CO_2_. Sub-confluent cells were transiently transfected for 12 h using Lipofectamine 2000 (Invitrogen) 24–48 h prior to experimentation.

### Primary Hepatocyte Isolation

Primary hepatocytes were isolated according to our previously published protocol [19].

### Protein Turnover Assays

Cycloheximide (CHX, 0.1 µg/µl) in fresh media was added to wells at the beginning of an experiment. Where indicated, 100 µM chloroquine (in H_2_O) or 10 µM MG-132 (in DMSO) was added 2 h prior to addition of CHX and maintained in the media throughout the end of the experiment. To biotinylate surface proteins, cells were washed on ice three times with cold PBS and incubated with freshly made maleimide-PEG_2_-Biotin (ThermoScientific) in PBS for one hour. Excess biotin was washed away three times in cold PBS with 1 mM DTT. Protein was extracted as below. Equal volumes of protein extract and streptavidin agarose slurry (Pierce ThermoScientific) were mixed and washed three times. Samples were rotated overnight at 4°C, washed three times, and mixed with a volume of SDS-Laemmli buffer. The supernatant was analyzed by SDS-PAGE.

**Figure 2:**
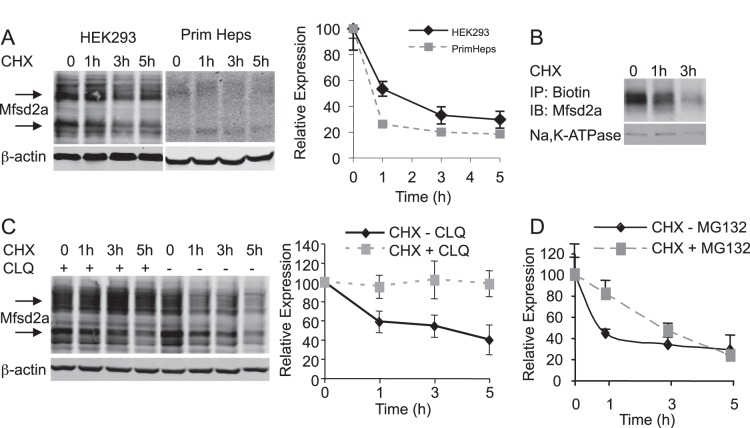
MFSD2A is rapidly turned over from the cell surface via the lysosome. *A,* MFSD2A turnover as a function of time in cycloheximide-treated (CHX) HEK293 cells transfected with mouse Mfsd2a and primary hepatocytes from fasted mice. Time indicates duration of CHX treatment. Graph quantifies MFSD2A expression. *B,* Turnover of plasma membrane MFSD2A in Mfsd2a-transfected HEK293 cells. Cells were incubated with CHX for the indicated time period and then treated with membrane-impermeable maleimide-PEG_2_-Biotin. Protein homogenates were immunoprecipitated with streptavidin beads, prior to Western blot analysis for plasma membrane localized MFSD2A. *C and D,* Mfsd2a-transfected HEK293 cells were treated with CHX as in panel *A*, with or without (*C*) chloroquine (CLQ) or (*D*) MG-132 (Western blot not shown). Protein extracts were analyzed by Western blot for MFSD2A. Graphs quantify MFSD2A expression. β-actin in *A* and *C* and Na-K-ATPase in *B* served as a loading control. Quantification represents experiments in triplicate, displayed as mean ± SD.

### Western Blotting

Mouse tissues were ground with a dounce homogenizer on ice in RIPA buffer with EDTA-free cOmplete protease inhibitor cocktail (Roche). Cell cultures were washed with cold PBS and scraped with cold RIPA buffer. Protein concentration was measured with a Bradford assay (BioRad). Protein samples (40 µg protein/lane) were separated by SDS-PAGE, transferred to nitrocellulose membranes (Bio-Rad), and incubated overnight with primary antibodies. Protein bands were quantified from infrared-linked secondary antibodies using an Odyssey infrared scanner (Li-Cor).

**Figure 3:**
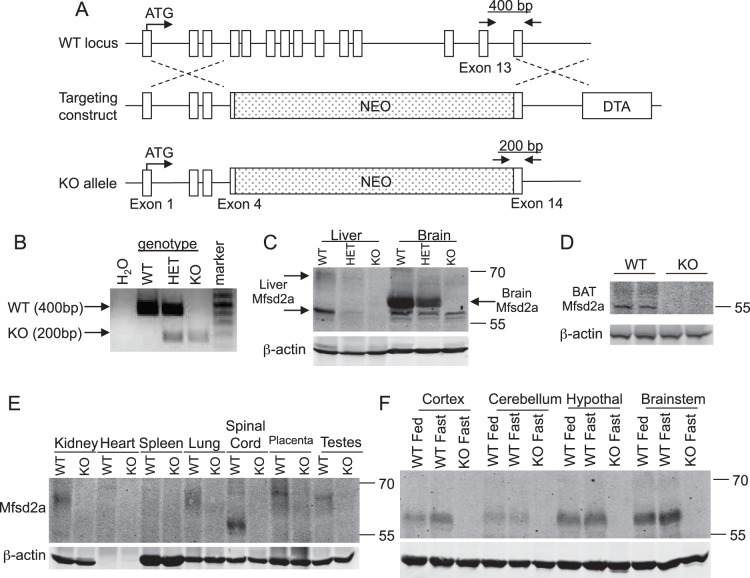
Generation of an Mfsd2a KO mouse. *A,* Schema for conventional Mfsd2a knockout where exons 4–13 were replaced with a neomycin resistance cassette (NEO). *B,* PCR genotyping of WT, HET and KO mice with primers indicated in *A*. *C,* Western blots of MFSD2A expression in liver and brain from fasted WT, HET, and KO male mice. *D,* Western blot of MFSD2A in liver of WT and KO mice exposed to 4°C. *E,* Western blot analysis of MFSD2A expression in indicated tissues from fasted WT and KO mice. *F,* Western blot of MFSD2A expression in the indicated brain regions in WT fed, WT fasted, and KO fasted mice. β-actin is the loading control in panels *C–F*. Note: lack of β-actin in heart sample in *D* is expected based on actin isoform expression.

**Figure 4:**
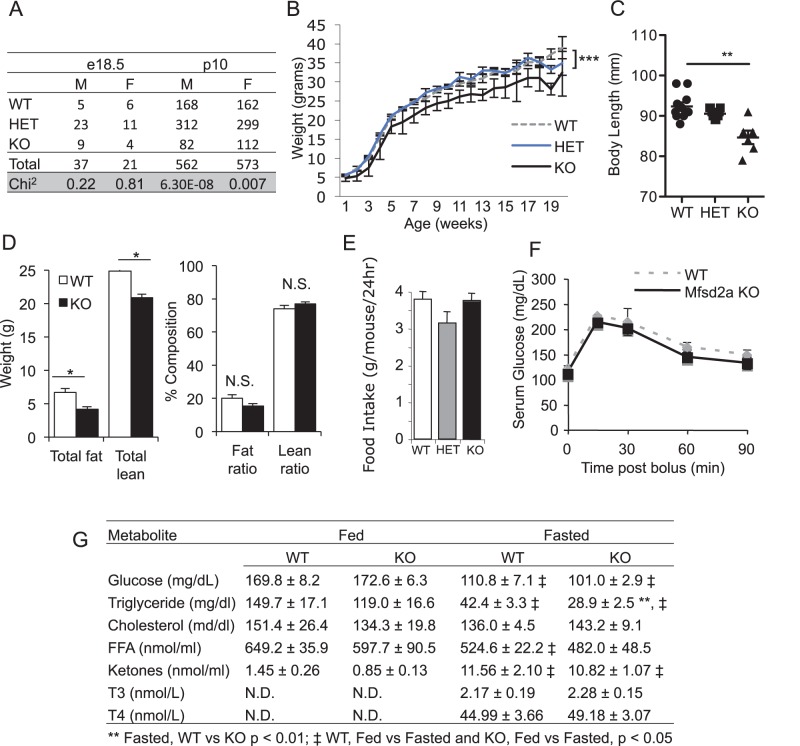
Mfsd2a KO mice are smaller and leaner than WT littermates, with reduced serum TG. *A,* Number of genotype obtained for male and female day e18.5 embryos (n = 7 litters, 62 embryos) and post-natal day 10 pups (n = 156 litters, 1135 pups). Chi^2^ analysis gives the probability that the observed ratios are Mendelian. *B,* Growth curves of weekly weights of offspring from heterozygous intercrosses for all three genotypes (average of n = 30 per genotype). Body weights between wildtype and KO mice are significantly different at all time points by week 2. *C,* Scatterplot of body lengths indicating average (from nose-to-anus) of WT, HET and KO littermates (n = 6–11). *D,* Absolute (left panel) and relative (right panel) adiposity of KO and WT littermates (n = 4–7) was determined by EchoMRI. *E,* Food intake per mouse, per 24 h (males, 14–16 weeks of age, n>5 per genotype). *F,* GTT of 14-week old chow-fed WT and KO males after overnight fast (n = 4). *G,* Metabolites and thyroid hormone from WT and KO male littermates, fed or post-18–24 h fast as indicated (n>4). Data are average ± SEM, * p<0.05, ** p<0.01, *** p<0.001, N.S. = not significant, N.D. = not determined.

**Figure 5:**
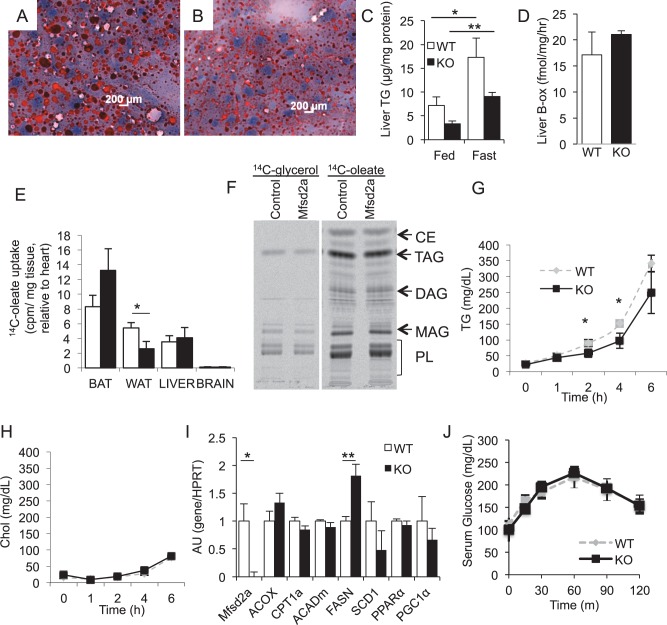
Mfsd2a KO mice have decreased liver TG with normal liver metabolism. Oil red-O histology of fasted *A,* WT and *B,* KO liver illustrates neutral lipid stores. *C,* Quantification of TG extracted from fed and fasted WT and KO livers. *D,* Assay of β-oxidation in liver homogenates from fasted WT and KO littermates (n = 3–4 per genotype, data representative of three independent experiments). *E, In vivo* uptake of ^14^C-oleate in indicated tissues from WT and KO mice, normalized to heart uptake (n = 3 per genotype). *F,* TLC of lipid extract illustrating *in vitro* incorporation of ^14^C-glycerol and ^14^C-oleate by control and Mfsd2a-transfected HEK293 cells (n = 3). CE, cholesterol ester; M/D/TAG, mono-, di-, tri-acylglycerol; PL, phospholipids. *G-H, In vivo* VLDL production was assayed by measuring *G,* serum TG and H, cholesterol in fasted WT and KO mice (n>5 per genotype) following treatment with LPL-inhibitor F-127. *I,* Real-time PCR analysis of PPARα targets and fatty acid synthesis genes from fasted WT and KO liver (n = 3–4). *J*, Pyruvate tolerance test of 12–14-week old chow-fed WT and KO littermates after an overnight fast (n = 6). Data are displayed as mean ± SEM. * p<0.05, ** p<0.01.

### Animal Models

A conventional KO mouse model was engineered following standard BAC recombineering protocols [20]. BAC RP23-306M6 was obtained from Children’s Hospital Oakland Research Institute. The engineered targeting vector replaced exons 4 through 13 with a PGK-Neomycin cassette, available from the NIH Division of Technology Development and Transfer Office. The vector was introduced into embryonic stem cells, and gene-targeted cells were implanted in C57BL/6J surrogate mothers. Mfsd2a KO mice used in these studies were backcrossed onto C57BL/6J for 5–10 generations. Gcgr KO mice, also on a C57BL/6J background, were previously described [21]. PPARα KO mice (Jackson Laboratory) were previously described [22]. Genotyping was performed on genomic DNA extracted from tail tips, using forward primer Mfsd2a TVP 1F 5′-GGCTTCAGGCACCCTGGCAAGTCCTAG-3′ and reverse primers PGK-R-TVP 5′-GCTTGGCTGGACGTAAACTCCTCTTCAGACC-3′ and Mfsd2a Intron 13 5′-GCCCTGTGTTCTAGCCACAACCTGTATTCTG-3′. Sex determination of neonates was performed as previously published [23].

### Animal Studies

Experimental protocols were approved by the Albert Einstein College of Medicine Institutional Animal Care and Use Committee. Mice were maintained on a 12 h light cycle on breeder chow (LabDiet #5058) or 60 kcal% high-fat diet (Research Diets, Inc. #D12492) as indicated. In all instances, “fasted” indicates an overnight (18–24 h) fast with *ad libitum* access to water. For the indicated studies, mice were weighed weekly. For glucose measurements, blood was collected from the tail vein and measured using a glucometer (Accu-Check Compact, Roche). For tolerance tests, overnight fasted mice were injected with bolus glucose (1 g/kg i.p.) or pyruvate (2 g/kg i.p.). Serum obtained by retro-orbital bleed was analyzed using colorimetric assays for total cholesterol and triglyceride (Infinity), ketone bodies (Stanbio), and non-esterified fatty acids (Wako). Total T3 and T4 levels were determined by RIA (MP Biologicals). Metabolic studies were conducted in a CLAMS (Columbus Instruments) indirect calorimetry system to obtain measures of oxygen consumption, carbon dioxide production, and physical activity. Mice acclimated to individual chambers for 2 days, followed by 4 days of data collection (48 h *ad libitum* feeding, 24 h fasting, and 24 h *ad libitum* refeeding). Data were normalized to lean body mass. Body mass was assessed with EchoMRI (Houston, TX). Tissue lipids were extracted from the volume of 1 mg protein (homogenized in PBS) with two volumes of 2∶1 chloroform:methanol. The organic phase was dried under nitrogen, resuspended in 1% Triton X-100, and quantified as above.

### VLDL Synthesis Assay

Mice were fasted for 4 h to clear chylomicrons from circulation, and injected with filter-sterilized Plurionic F-127 (1 mg/g BW i.p.) in PBS [24], [25]. Blood was collected immediately prior to injection (t = 0) and thereafter hourly up to 4 h, and analyzed for serum TG and cholesterol concentrations as described above.

### *In vivo* Fatty Acid Uptake Assay

Fasted animals received i.p. injections of PBS with 1 mM oleate/BSA and 2 µCi [14C]-oleate/BSA. Mice were sacrificed after 1 h, perfused with PBS, and tissues were removed, weighed, and snap-frozen. Whole tissue was ground in PBS, scintillation counted, and normalized as both as cpm per g wet weight and per mg protein.

### Fatty Acid Re-Esterification Assay

Transfected HEK293 cells were incubated with either 0.5 µCi [14C]-glycerol or 100 µM oleate/BSA with 0.4 µCi/µl [14C]-oleate/BSA in KRPH buffer (5 mM NaH_2_PO_4_, 20 mM HEPES, 1 mM MgSO_4_, 1 mM CaCl_2_, 136 mM NaCl, 4.7 mM KCl) for 3 h at 37°C. Cells were washed with 100 µM oleate/BSA in PBS, air dried, and lipids were extracted with 3∶2 hexane/isopropanol. Thin layer chromatography (TLC) separation of extracted lipids was performed as previously described [26]. Signals were normalized to total protein extracted from cells with 0.1 N NaOH/0.1% SDS solution.

### Measurement of β-oxidation in Liver Homogenate

As modified from [27], perfused liver was excised, weighed, and immediately homogenized in ice-cold buffer (0.25 M sucrose, 1 mM Tris-HCl, 1 mM EDTA pH 7.4 with protease inhibitors). 10 mg worth of liver tissue was added to 4 volumes (∼200 µl) of reaction buffer (150 mM KCl, 10 mM HEPES, 0.1 mM, 1 mM KH_2_PO_4_, 10 mM MgCl_2_-6H_2_O, 5 mM malate, 1 mM L-carnitine, 5 mM ATP, pH 7.2 with 50 µM Palmitate/BSA and 1 µCi of [3H]-palmitate/BSA per rxn) and incubated at 37°C for 1 h. Reaction was stopped with addition of 200 µl 0.6 M perchloric acid and the media was cleared of debris by centrifugation at 2,000×g for 5 min. One volume of radioactive media was mixed with nine volumes of 20 mM Tris-HCl, pH 7.5 with 10% w/v activated charcoal suspension and rotated for 30 min at room temperature to remove free fatty acids from media. The sample was centrifuged at 13,000×g for 15 min, and 200 µl of supernatant was scintillation counted to measure tritiated water. FAO was calculated as (CPMs sample – CPMs blank) and converted to nmols palmitate per mg tissue.

### *In vivo* and *ex vivo* Lipolysis Assays

For measurement of lipolysis *in vivo*, blood was collected with non-heparinized capillary tubes for baseline measurement prior injecting mice with 1 mg/kg BW i.p. CL316243 (Sigma). Blood was collected 15 min later for serum NEFA measurements [28]. For *ex vivo* lipolysis assays, adipose tissue explants (∼20 mg) from epididymal fat pads were incubated in Krebs-Ringer buffer (12 mM HEPES, 121 NaCl, 4.9 mM KCl, 1.2 mM MgSO_4_ and 0.33 mM CaCl_2_) with 3.5% fatty acid-free BSA and 3 mM glucose (KRB), with or without 200 nM isoproterenol (Sigma) at 37°C for 2 h and measured for glycerol (Sigma) and fatty acid (Wako) production as previously published [29].

### Brown Adipose Function

β-oxidation was measured from bilateral BAT depots isolated from sacrificed mice, trimmed clean of WAT, weighed, and incubated in KRB with 50 µM [9,10-3H]-palmitate/BSA (0.4 µCi/ml) and with or without 200 nM isoproterenol at 37°C for 2 h. At the endpoint, tissue was snap-frozen in liquid nitrogen, and the media was clear of debris by centrifugation at 2,000×g for 5 min. The media was washed with charcoal as above to remove free radioactive palmitate and scintillation counted.

### Cold Tolerance Test

Cold-induced thermogenesis was evaluated by exposure to 4°C for at least 2 h. Animals were fasted, with *ad libitum* access to water, and housed individually without bedding. Rectal temperature was monitored with a Physiotemp IT-23 thermocouple microprobe. BAT mitochondrial content was assessed by western for Tom20 and CoxIV protein expression, as well as by the ratio of UCP1 DNA copy number to mitochondrial CoxII DNA assessed by real-time PCR.

### Histology

Adipose samples were fixed in 10% formalin for 24 h followed by 70% ethanol, prior to paraffin embedding, sectioning, and staining by hematoxylin and eosin (H&E). Liver was frozen in OCT medium for Oil Red-O staining.

### Neurologic Assessment

Analysis of motor coordination and balance was objectively measured by performance on an accelerating rotarod. Mice were trained and tested with three trials per day for 3 days starting at a speed of five rotations per minute, with a 0.1 rpm/sec increase for no longer than 5 min, measuring latency to fall-off.

### mRNA Transcript Analysis

Mouse liver and BAT RNA was isolated with Trizol, and cDNA was synthesized using a SuperScript III first-strand cDNA synthesis kit (Invitrogen). Quantitative real-time PCR was preformed using SYBR Green on a 7500 Fast system (Applied Biosystems). Relative quantification of each target was normalized to HPRT for liver and ribosomal 18S for BAT. Primer (Invitrogen) sequences are available on upon request.

**Figure 6:**
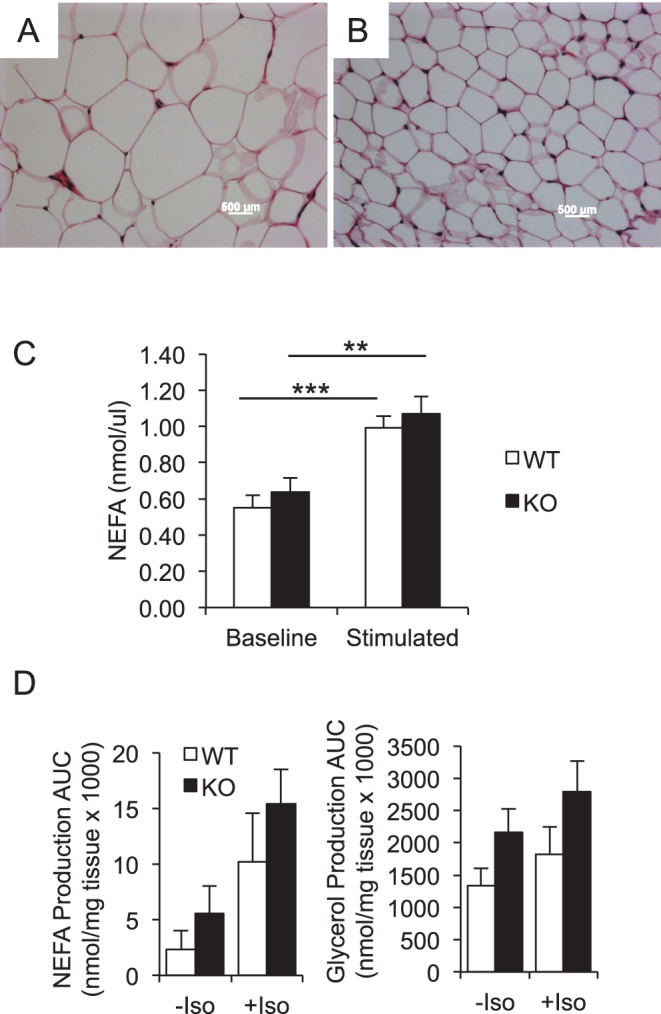
Mfsd2a KO mice have decreased adiposity but normal lipolysis. *A,* WT and *B,* KO WAT depots stained with H&E. *C,* NEFA measurements after a 4 h fast (baseline) and 15 min after an injection of adrenergic agonist CL316243 (stimulated). *D,* Lipolysis was assayed by measuring NEFA (left panel) and glycerol (right panel) production over two hours, with and without isoproterenol (ISO) stimulation, from WAT explants isolated from fasted WT and KO mice. Data are displayed as mean ± SEM. ** p<0.01, *** p<0.001.

**Figure 7:**
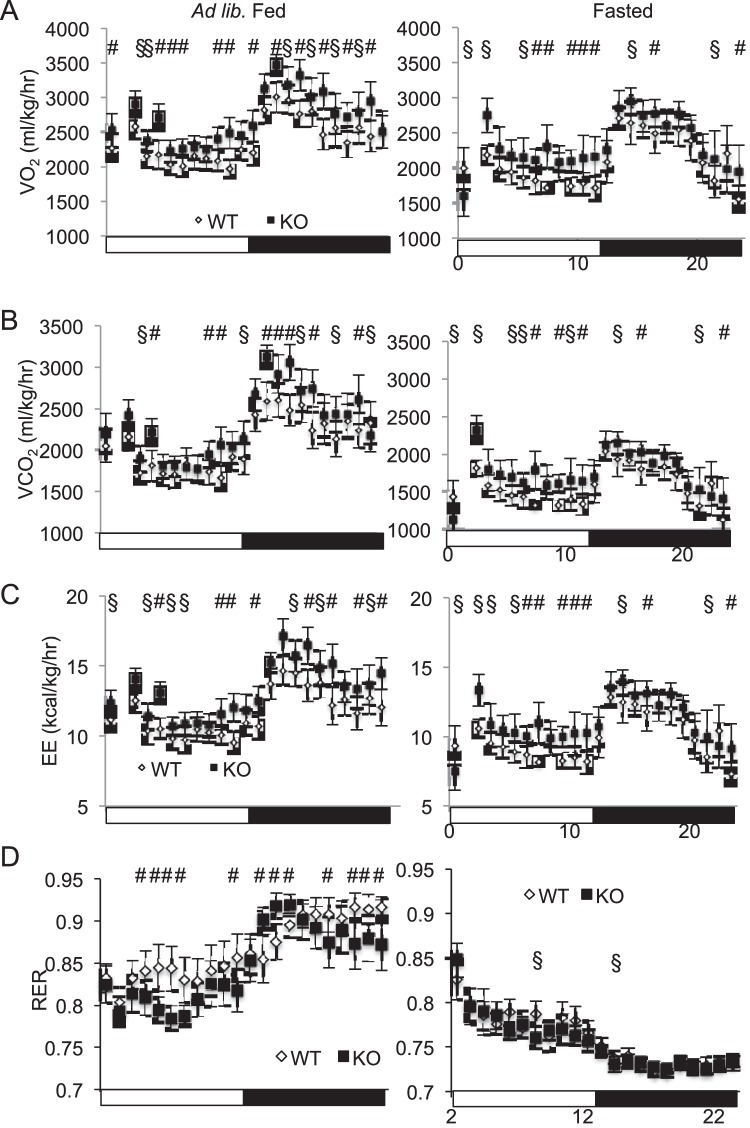
Mfsd2a KO mice have increased energy expenditure. *A–D,* Indirect calorimetric analysis of male WT (white bars) and KO (dark bars) mice (n = 4, 12–14 weeks of age) under *ad libitum* fed (left column) and fasted (right column) conditions for *A*, VO_2_, *B,* VCO_2_, *C*, energy expenditure (EE), and *D*, respiratory quotient (RER). § p<0.05, # p<0.001.

**Figure 8:**
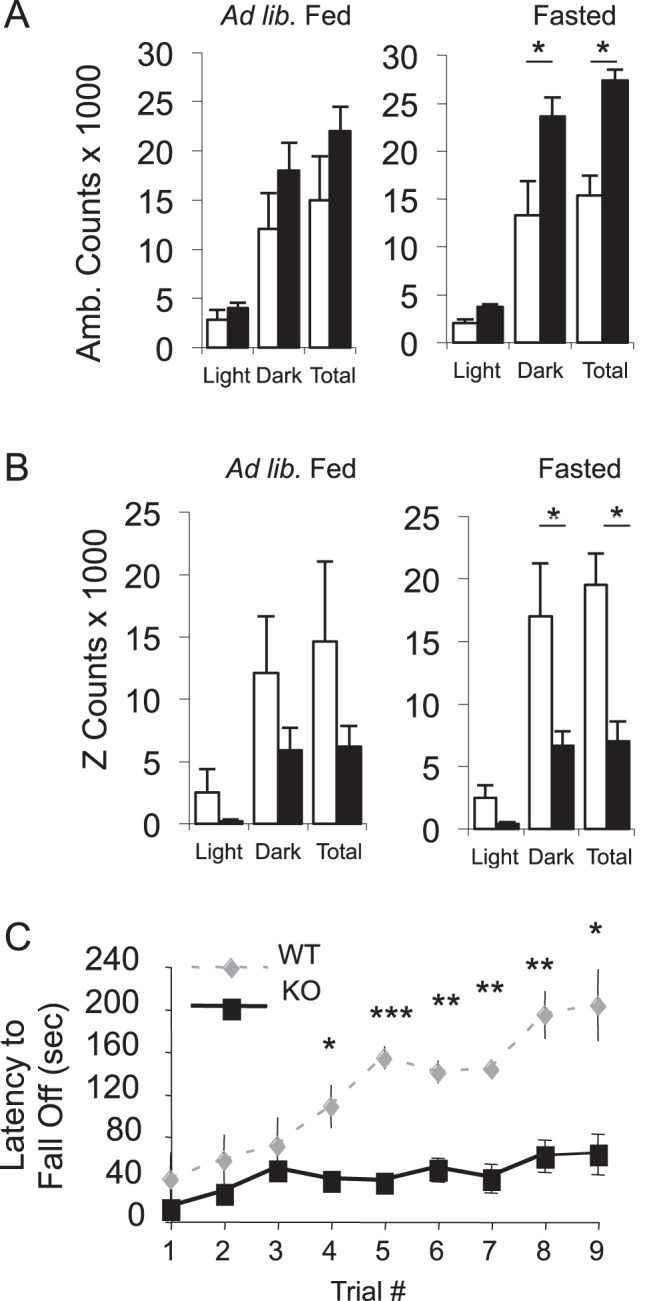
Mfsd2a KO mice have increased, ataxic movement. *A,* X-Y movement and *B*, Z-axis movement under *ad libitum* fed (left column) and fasted (right column) conditions for WT and KO cohorts from Fig. 7. *C,* Rotarod analysis to quantitatively assess motor coordination in WT and KO littermates (n>7). Data are displayed as mean ± SEM. * p<0.05, ** p<0.01, *** p<0.001.

## Results

### Fasting-induced Expression of MFSD2A Requires PPARα and Glucagon Signaling

In order to study the regulation and expression of MFSD2A, a polyclonal antibody was raised against the c-terminus of mouse MFSD2A. We verified that mouse MFSD2A has two glycosylation sites at Asn-221 and Asn-231 (homologous to the sites Asn-217 and Asn-227 in human MFSD2A), and is localized to the plasma membrane (data not shown), as previously demonstrated [18], [30]. In mouse liver and BAT, MFSD2A is expressed as two major species – a predicted non-glycosylated 59 kDa form and a glycosylated ∼70 kDa form (Fig. 1A). The brain expresses only one ∼65 kDa species of MFSD2A (Fig. 1A).

*Mfsd2a* mRNA has previously been shown to be upregulated in liver and BAT by fasting and in BAT by cold exposure [17]. Expression of MFSD2A was undetectable in the liver of *ad libitum* fed wildtype mice, but significant induction was observed in livers from fasted wildtype mice (Fig. 1A). Contrary to the robust mRNA induction of *Mfsd2a* in BAT [17], the induction of MFSD2A protein by fasting was modest (Fig. 1A). Refeeding of fasted mice resulted in a return of MFSD2A protein to baseline in both liver and BAT. Expression of MFSD2A in whole brain was constitutive and did not appear to be fasting-regulated (Fig. 1A). Expression is absent in white adipose tissue (WAT) (Fig. 1A).

**Figure 9:**
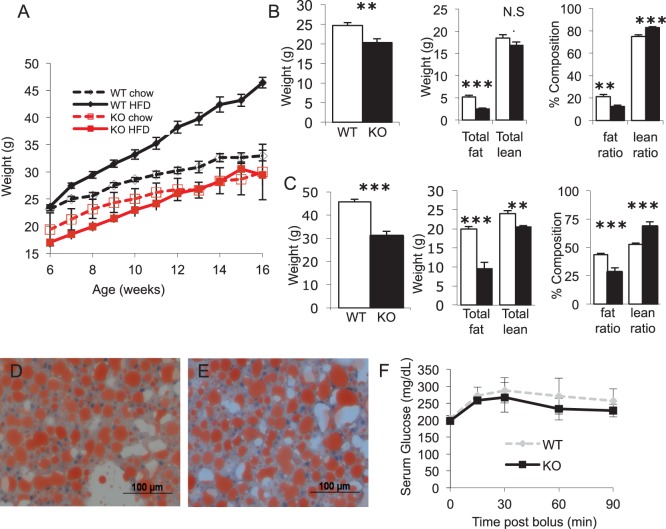
Mfsd2a KO mice develop glucose intolerance but are resistant to diet-induced obesity. *A,* Growth curves for WT and KO male mice fed chow or high-fat diet (HFD) starting at 6 weeks of age (n = 4–8 per genotype). *B and C*, Average body weight (left), absolute adiposity (center), and relative adiposity (right) for WT (open bar) and KO (closed bar) cohorts fed *B,* 10 days and *C,* 10 weeks of HFD. *D,* WT and *E,* KO livers stained with ORO for neutral lipid following 10 weeks of HFD. *F,* GTT after overnight fast of WT and KO males fed HFD for 10 weeks (n = 3–4 mice per genotype, data representative of two independent experiments). Data are displayed as mean ± SEM. * p<0.05, ** p<0.01, *** p<0.001, N.S. = not significant.

With a focus on MFSD2A regulation in liver, we sought to determine the transcriptional pathways that mediate its nutritional regulation. Since many fasting-induced genes are regulated by the nuclear hormone receptor PPARα [31], we used PPARα KO mice to genetically test that PPARα was necessary for the fasting-induced expression of MFSD2A. Fasted PPARα KO mice failed to induce MFSD2A compared to wildtype controls (Fig. 1B). Given that glucagon plays a dominant role in regulating liver metabolism during a fast [32], [33], and that glucagon signaling has been shown to synergize with PPARα for induction of genes in the liver [34], [35], we explored the possibility that glucagon signaling was additionally required for fasting induction of MFSD2A. Indeed, fasted glucagon receptor KO mice failed to induce MFSD2A in the liver (Fig. 1C). Together, these findings indicate a dependence on both PPARα and glucagon signaling for upregulation of MFSD2A in response to fasting.

**Figure 10:**
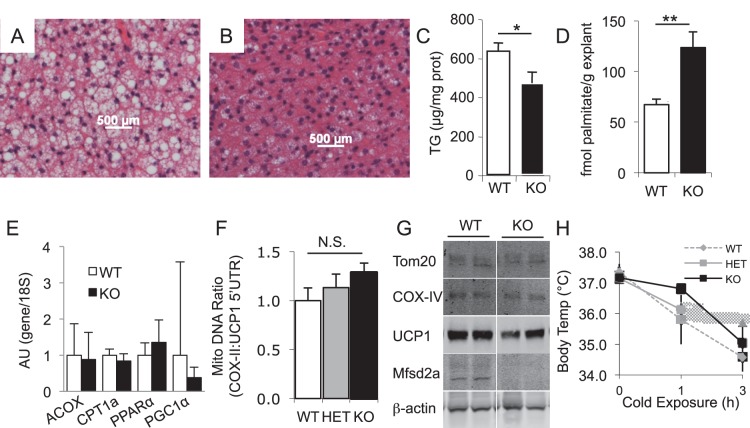
Mfsd2a KO mice have increased BAT β-oxidation. H&E staining of BAT depots from fasted *A,* WT and *B,* KO mice. *C,* Quantification of TG from WT and KO BAT (n>7 per genotype). *D,* Assay of β-oxidation in BAT explants from fasted WT and KO littermates (n = 4). *E,* Real-time PCR analysis of PPARα and PPARα targets in BAT from fasted WT and KO mice. *F,* Quantification of mitochondrial genome numbers from the ddCT of real-time PCR signal for COX-II (mitochondrial) to UCP1 (nuclear) DNA from BAT (n>4). *G,* Western blots probed for BAT mitochondrial membrane proteins Tom20, COX-IV and UCP1 from fasted WT and KO mice (n = 3). *H,* Body temperature measurements to assess cold-induced (non-shivering) thermogenesis in cohorts of fasted WT, HET, and KO mice (n = 3–4 per genotype) exposed to 4°C. Data are displayed as mean ± SEM. * p<0.05, ** p<0.01.

Since we observed loss of MFSD2A protein expression upon refeeding (Fig. 1A), we sought to determine the kinetics of MFSD2A down-regulation following refeeding. Wildtype mice were fasted for 24 h from dark cycle to dark cycle, and followed over a time course of 9 h of *ad libitum* refeeding at the onset of the second dark cycle (Fig. 1D). Expression of MFSD2A was expectedly high at the end of 24 h fast/start of refeeding (t = 0) and during the subsequent first hour of access to food (t = 1 h). By 3 h of refeeding, MFSD2A was undetectable, indicating rapid down-regulation. Interestingly, by t = 9 h of refeeding and approaching the beginning of the next light cycle, protein expression had begun to increase.

Our findings indicate that MFSD2A protein expression is tightly regulated by fasting and refeeding, suggesting that in addition to transcriptional control [17], MFSD2A protein turnover might be important to controlling hepatic MFSD2A levels. To test this idea, we determined the half-life of MFSD2A protein expressed from a strong promoter in HEK293 cells, as well as turnover of endogenous MFSD2A from primary hepatocytes. HEK293 cells expressing mouse MFSD2A and mouse primary hepatocytes were treated with cycloheximide to block ongoing translation, and MFSD2A protein levels were quantified over a time course. Degradation of MFSD2A occurred with a half-life of approximately 60 min in HEK293 cells and primary hepatocytes (Fig. 2A). Biotin labeling of the cell surface of HEK293 cells indicated that MFSD2A turnover occurred from the plasma membrane with a similar rate as determined in whole cell lysates (Fig. 2B). In addition, inhibition of lysosomal function using chloroquine, but not the proteosome inhibitor MG-132, inhibited MFSD2A turnover in the presence of cycloheximide (Fig. 2C–D), indicating that MFSD2A turnover from the plasma membrane results in its degradation in lysosomes.

### Mfsd2a KO Mice are Leaner, with Less Serum and Tissue Triglyceride

Based on our finding that fasting induction of MFSD2A in the liver is dependent on PPARα and glucagon signaling, we hypothesized that MFSD2A plays a physiological role in lipid metabolism in response to a fast. To test this hypothesis, we generated a conventional gene-targeted mouse model of MFSD2A deficiency (Fig. 3A). Offspring derived from Mfsd2a heterozygous intercrosses resulted in the recovery of all Mfsd2a genotypes (Fig. 3B). MFSD2A protein levels in the livers and brains from fasted mice displayed dependency on gene-dosage (Fig. 3C). While fasting did not strongly induce MFSD2A in BAT (Fig. 1A), cold exposure of wildtype mice made brown adipose MFSD2A, which is absent in the KO littermates, easier to detect (Fig. 3D). We also observed low-level expression of MFSD2A protein in kidney, testes, spinal cord, and placenta. MFSD2A protein was not detected in heart, lung, spleen, or WAT (Fig. 3E). MFSD2A was expressed in multiple anatomical regions in the brain but was not induced by fasting (Fig. 3F). Based on the nutritional regulation of MFSD2A expression, we primarily focused on characterizing phenotypes within liver and BAT.

Mfsd2a heterozygous mice developed normally and were fertile. Divergence from Mendelian inheritance was observed at post-natal day 10 from heterozygous intercrosses indicating increased mortality of both male and female Mfsd2a KO mice (Fig. 4A). Normal Mendelian ratios were found at embryonic day 18.5, indicating that the loss of Mfsd2a KO genotype occurs early in postnatal life. By two weeks of age, male and female KO mice weighed significantly less than wildtype and heterozygous littermates (Fig. 4B, female data not shown). As seen in the growth curves, this weight difference is maintained and accentuated as the mice age. Additionally, the KO mice are shorter in length than wildtype littermates (Fig. 4C). In male KO mice, body adiposity as determined by EchoMRI was significantly decreased in absolute fat mass (Fig. 4D). Despite the differences in body size, measurement of food intake indicated that KO mice consumed similar amounts of food as their wildtype littermates (Fig. 4E). Glucose homeostasis, measured by glucose tolerance tests (Fig. 4F) and fed and fasting serum glucose (Fig. 4G), showed no difference between chow-fed wildtype and KO mice. KO mice showed a significant decrease in total fasting (20 hr fast) plasma triglycerides (TG) with no significant differences in fasting glucose, total cholesterol, non-esterified FA, and ketones (Fig. 4G). Free T3 and T4 thyroid hormone levels were normal in KO mice (Fig. 4G).

### Mfsd2a KO Mice have Normal Liver Physiology but Increased Energy Expenditure

Because fasting induces MFSD2A, and knockout mice exhibit reduced adiposity and serum TG, we wanted to determine whether an overnight fast of Mfsd2a KO mice would result in altered TG deposition in liver. It is well established that overnight fasting of mice results in significant accumulation of liver triglyceride [36]–[38]. Oil red-O stained liver sections from fasted wildtype and Mfsd2a KO mice indicated a significantly reduced accumulation of neutral lipid in livers of Mfsd2a KO mice (Fig. 5A vs. 5B). We confirmed this observation by enzymatic quantification of liver TG (Fig. 5C). However, the fold-increase in liver TG from fed to fasted state in both wildtype littermates and Mfsd2a KO mice was not significantly different (WT 2.4-fold vs. KO 2.7-fold, p = 0.33).

Among the explanations for decreased TG load within the liver are an increase in β-oxidation, decreased FA uptake, decreased TG biosynthesis, and increased VLDL production, or inadequate substrate availability due to changes in lipolysis or overall decreased fat mass. Liver β-oxidation in fasted Mfsd2a KO mice was not significantly different than wildtype littermates (Fig. 5D). No significant differences were measured for the uptake of ^14^C-oleate *in vivo* into liver (Fig. 5E). This is consistent with the finding that expression of MFSD2A in HEK293 cells did not affect fatty acid or glycerol incorporation into TG and phospholipids (Fig. 5F). Interestingly, WAT from KO mice showed decreased oleate uptake *in vivo* relative to wildtype, which is likely a secondary effect of Mfsd2a deficiency because Mfsd2a is not expressed in WAT (Fig. 1A). VLDL production was significantly decreased at 2 h and 4 h of measuring VLDL production in KO relative to wildtype mice (Fig. 5G–H). Note that KO and wildtype had similar plasma TG after a 4 hr fast (Fig. 5G), but KO levels were significantly decreased after a 20 hr fast (Fig. 4G). Additionally, PPARα targets in liver involved in FAO, as well as genes important for fatty acid synthesis and lipid signaling, were largely unchanged, with the exception of increased FASN expression (Fig. 5I). Lastly, in line with normal glycemia in KO mice, decreased hepatic fat in KO mice did not alter hepatic glucose production, as determined by pyruvate tolerance test (Fig. 5J).

Histology of WAT revealed smaller adipocytes in KO tissue (Fig. 6A vs. 6B), consistent with reduced fat mass (Fig. 4D). We next tested if decreased fat mass in Mfsd2a KO mice was due to changes in adipocyte lipolysis. *In vivo* stimulation of lipolysis indicated no significant difference in KO mice (Fig. 6C), suggesting that increased sympathetic activity in KO mice is not the cause for decreased adipocyte size and mass. *Ex vivo* lipolysis assays using adipose tissue explants confirmed these *in vivo* findings that KO WAT exhibits comparable lipolysis to wildtype depots (Fig. 6D). Taken together, the data indicate that decreased liver TG in Mfsd2a KO mice in fed and fasted state is likely due to decreased whole body adiposity and not due to enhanced FAO, decreased FA uptake capacity, or increased VLDL secretion.

We examined whether increased whole-body energy expenditure could explain the phenotype of leaner body composition, and thus decreased liver TG. To do so, we measured energy expenditure using indirect calorimetry. KO mice had significantly increased VO_2_, VCO_2_, and energy expenditure, particularly during the dark cycle during *ad libitum* feeding, and light cycle in the fasted state (Fig. 7A–C). RER was increased in fed KO mice, though wildtype and KO mice reacted similarly to fasting, indicating that the KO mice retained overall metabolic flexibility for substrate utilization (Fig. 7D).

No statistically significant difference in movement was seen among *ad libitum* fed mice (Fig. 8A). Interestingly, ambulation (activity in the X-Y plane) was significantly higher in the fasted KO compared to wildtype littermates, particularly during the dark, food-seeking cycle (Fig. 8A). Conversely, rearing (Z-axis movement) was significantly depressed in fasted KO mice (Fig. 8B). To confirm this observation, we conducted rotarod testing to determine whether decreased rearing indicated a defect in motor coordination. Performance of KO mice on a rotarod indicated that KO mice had a pronounced ataxia (Fig. 8C).

Since Mfsd2a KO mice exhibited increased energy expenditure, we hypothesized that KO mice would be protected from high fat diet-induced weight gain. To test this hypothesis, we challenged Mfsd2a KO and wildtype littermates with a 60% kcal fat diet (HFD) for 10 weeks, starting at six weeks of age. Throughout the experimental time-course, male Mfsd2a KO mice were protected from diet-induced obesity, weighing similar to KO mice fed chow diet and significantly less than wildtype HFD animals (Fig. 9A). Measurements of body composition of the cohorts at 10 days of HFD (Fig. 9B) and 10 weeks of HFD (Fig. 9C) confirm the lean nature of the KO animals. Despite the protection from obesity, by 10 weeks of HFD feeding, wildtype and KO mice had similar levels of hepatosteatosis (Fig. 9D vs. 9E) and were similarly glucose-intolerant (Fig. 9F).

### Mfsd2a KO Mice have Increased FAO in BAT

In light of data suggesting a role for MFSD2A in BAT activation in the setting of cold-induced thermogenesis and adrenergic stimulation [17], we used the Mfsd2a KO animals to assess BAT function. We observed a similar histologic phenotype to the liver in KO BAT, where KO samples appeared densely eosinophilic, with fewer multilocular lipid droplets compared to wildtype (Fig. 10A vs. 10B). Total TG content was significantly decreased (Fig. 10C). Importantly, the oxidative capacity of the subscapular BAT depots, measured by *ex vivo* oxidation assays, was significantly higher in KO BAT relative to wildtype BAT (Fig. 10D). As other oxidative genes showed no changes in message level (Fig. 10E), and there was no increase in mitochondrial number (Fig. 10F–G), this may indicate a role for MFSD2A in regulation of BAT oxidation. Lastly, Mfsd2a KO mice, regardless of the reduced BAT TG content, were able to defend their body temperature during cold challenge as well as their fasted wildtype littermates (Fig. 10H).

## Discussion

In this study, we have determined that MFSD2A plays roles in lipid metabolism, body growth, and motor coordination. MFSD2A is doubly glycosylated and localized to the cell surface, consistent with findings regarding other members of the Major Facilitator Superfamily [17], [18], [30]. Previous data have indicated circadian control in Mfsd2a mRNA expression [17], [39], and suggests that downregulation of expression might be under the control of RORα1 and RORγ1 [17]. Here we extend these findings and have shown that MFSD2A protein expression during fasting is dependent on PPARα and glucagon signaling (Fig. 1B, C), and exhibits rapid turnover in the liver following refeeding (Fig. 1D). MFSD2A protein expression is tightly regulated with a half-life of less than 60 min in cell culture (Fig. 2A). Of note, expression in the brain is constitutive, ubiquitous, and not fasting-induced as in liver or BAT (Fig. 3F).

Mfsd2a KO mice are leaner and smaller than wildtype littermates and consistently have less TG in liver (Fig. 5C), BAT (Fig. 10C) and serum (Fig. 4G). The overall reduced adiposity and body size could represent a developmental defect or chronic adaptation to loss of MFSD2A. This idea is supported by the observations that the fold increase in TG accumulation in livers of Mfsd2a KO mice following a fast was similar to wildtype littermates, KO mice have unchanged liver fatty acid oxidation, and reduced VLDL production relative to wildtype (Fig. 5).

Alternatively, MFSD2A KO mice have a significant increase in whole-body energy expenditure (Fig. 7C), particularly during the light cycle of fasted mice when MFSD2A would be induced. While liver fatty acid metabolism appeared normal and unable to account for this expenditure, BAT activity was significantly increased in Mfsd2a KO mice (Fig. 10D). BAT activity in rodents has been correlated with regulation of body weight in response to increased caloric intake [40]–[42]. Moreover, increased brown adipose FAO could also explain decreased plasma triglycerides and potentially liver triglycerides [43]. The observation that MFSD2A induction by fasting requires PPARα and glucagon signaling would have intuitively suggested that MFSD2A, like many other PPARα and glucagon targets, plays a role in activating FAO. However, our findings here do not support this conclusion, since liver FAO was unchanged and BAT FAO was increased in KO mice. In short, the phenotype of the MFSD2A deficient mouse resembles neither the PPARα nor Gcgr KO models [10], [21], [22], [35]. Our data more likely support the conclusion that MFSD2a does not regulate PPARα or glucagon signaling, but rather the increased BAT FAO is an adaptive response to loss of neuronal MFSD2A. Indeed, the brain is the primary tissue of expression of Mfsd2a and efferent signals from the brain are known to regulate BAT activity [11], [44]–[47].

In addition to increased BAT oxidation, Mfsd2a KO mice exhibited significantly increased voluntary movement, but with surprisingly decreased motor coordination (Fig. 8A and 8C) [48]–[50]. Both the motor deficits and BAT activity strongly suggest a central effect of MFSD2A action. In addition to the neurologic changes observed, Hogenesch and co-workers [39] recently determined that mouse Mfsd2a is primarily under central nervous system (CNS) clock control for circadian expression, and not liver clock control. Hogenesch and co-workers raise the intriguing idea that Mfsd2a serves to deliver nutritional signals to the liver clock. Whether the CNS- or liver-expressed Mfsd2a plays any role in circadian control of metabolism remains to be determined. Regardless, the combination of increased voluntary movement and increased BAT activity likely accounts for the increased energy expenditure, and thus the lean nature of the MFSD2A KO mice, including protection from diet-induced weight gain (Fig. 9A).

The small size and post-natal lethality of Mfsd2a KO mice are notable since MFSD2A is expressed in placenta, and human MFSD2A has been shown to be the receptor for the syncytiotrophoblast fusion factor syncytin-2 [18]. However, Heidmann and coworkers have demonstrated that mouse MFSD2A is not a receptor for mouse syncytin-A or -B, the murine syncytin homologs. Moreover, syncytin-A deficiency results in early embryonic lethality, while mice lacking syncytin-B experience late onset growth retardation but without any post-natal lethality as observed in Mfsd2a KO mice [51], [52]. It remains to be determined whether MFSD2A plays a role in placental development, and whether possible defects in placental function are responsible for the small post-natal size and mortality of Mfsd2a KO mice, as well as the metabolic changes reported here. The Mfsd2a KO model may yet provide interesting observations about the role of MFSD2A in placentation.

Clearly, identification of the physiological ligand(s) transported by MFSD2A will provide great insight into the physiological function of MFSD2A. Mfsd2a has limited homology to the bacterial disaccharide melibiose transporter [17], and may have the capacity to transport the bacterial-derived glycosylation inhibitor tunicamycin [30]. While we did not find MFSD2A capable of transporting glucose, fatty acids, pyruvate, ketones, or select amino acids (data not shown), and Sabatini and co-workers reported an absence of transport of melibiose [30], it is possible based on the suggested transport of tunicamycin, itself an acylated disaccharide, that the endogenous substrate may be a metabolically active glycolipid.

While MFSD2A expression is tightly regulated in the liver and BAT, it is ubiquitously expressed in the brain (Fig. 3F). Based on prominent hyperactivity (Fig. 8A) and ataxia (Fig. 8C), as well as the small body size (Fig. 4B–C) and early post-natal death (Fig. 4A) of Mfsd2a KO mice, it is likely that MFSD2A plays a profound role in brain metabolism. Only through future identification of its ligand(s) will these questions about the central role of MFSD2A be answered.
